# Knowledge and perception of antimicrobial resistance and antimicrobial stewardship among healthcare students in Nigeria

**DOI:** 10.4314/ahs.v23i4.22

**Published:** 2023-12

**Authors:** Idongesit L Jackson, Mary R Akpan, Grace O Adebayo

**Affiliations:** Department of Clinical Pharmacy and Biopharmacy, University of Uyo, Akwa Ibom State, Nigeria

**Keywords:** Antimicrobial resistance, antimicrobial stewardship, healthcare students, Nigeria

## Abstract

**Background:**

Assessment of knowledge and perception of healthcare students regarding antimicrobial resistance (AMR) and antimicrobial stewardship (AMS) would facilitate more effective education of these future prescribers.

**Objectives:**

To assess knowledge and perception of AMR and AMS among healthcare students in Nigerian universities.

**Methods:**

This was a questionnaire-based, cross-sectional survey of medical, nursing and pharmacy undergraduate students from November 2019 to January 2020, using both paper and electronic modes of self-administration.

**Results:**

A total of 335 students participated in the survey. Mean age of respondents was 23±3 years; 114 (34.4%) were in their 5th year of study. Most (78.9%) of the respondents agreed that widespread use of antimicrobials promotes AMR. Only 70 (21.1%) were aware that poor hand hygiene promotes AMR; 45.9% (42.7%, 37.3% and 57.7% for medicine, nursing and pharmacy respectively, p = 0.007) agreed that AMR is promoted by substandard quality of antimicrobials. Majority (94.3%) perceived AMR as a worldwide problem. Over half (60.8%) were not familiar with the term ‘antimicrobial stewardship’. Eleven (3.3%) and 122 (36.9%) rated their AMS knowledge as ‘very good’ and ‘poor’ respectively.

**Conclusions:**

Nigerian healthcare students had suboptimal knowledge of AMR and AMS. Current undergraduate healthcare curriculum should be reviewed to incorporate AMS principles.

## Introduction

The rise in antimicrobial resistance (AMR) poses a major societal threat with clinical, economic and developmental consequences including, prolonged illness, increased mortality and costs.^1^,^2^ This has resulted in a resort to older antimicrobials which have become increasingly in-effective.^3^ A myriad of factors contributes to the development of AMR including inadequate regulations, lack of knowledge towards best practices, online sales, misuse and overuse of antimicrobials in humans and animals, and suboptimal dosing.^4^,^5^

To mitigate the growing threat of AMR, the World Health Assembly, in 2015 adopted a global action plan on antibiotic resistance. The goals of the action plan, among others are to improve awareness and understanding of AMR through effective communication, education and training, strengthen the knowledge and evidence base through surveillance and research and optimise antimicrobial use in human and animal health.^6^

Antimicrobial stewardship (AMS) is an intervention^7^ that ensures optimal selection, dosage, and duration of treatment to achieve best clinical outcomes for the treatment or prevention of infections and minimize unintended consequences and development of resistance.^8^,^9^ Ultimately, the goal of AMS is to improve safety and conserve the available antimicrobials. Healthcare professionals and those in training therefore have a role in combating the increasing problem of AMR.^9^

Healthcare professionals, especially physicians, pharmacists and nurses are at the central role of prescribing and dispensing antimicrobials to patients. Thus, the next generation of healthcare professionals must be ready to take a more central role in ensuring appropriate use of available antimicrobials. Education and training of healthcare workers and students on AMS has been identified as an integral part of AMR containment activities.^10^,^11^ Although the inclusion of education on appropriate use of antimicrobials in the students' curricula and continuing education has been reported in different countries,^12^-^15^ there are limited data on the content and effectiveness of the education of healthcare students regarding appropriate antimicrobial prescribing and AMS in Nigeria. This study sought to assess knowledge and perception of AMR and AMS among healthcare students in Nigerian universities.

## Methods

### Study design, sampling and population

This was a cross-sectional, descriptive survey carried out among medical, nursing and pharmacy undergraduate students in Nigerian universities. Nigeria is divided into six geopolitical zones, namely North-Central, North-West, North-East, South-South, South-West, and South-East. At the time of data collection, there were 40, 26, and 21 universities in Nigeria offering undergraduate courses in medicine, nursing, and pharmacy, respectively. Medicine required six years of study whereas nursing and pharmacy required five; at the time, only one university offered a 6-year pharmacy degree. Twenty-three universities from the six zones were selected by convenience sampling. Sampling of respondents from each of the participating institutions was by convenience; however, respondents had to be third year undergraduate students or higher enrolled in pharmacy, medicine, or nursing programmes.

### Survey instrument and data collection

Questionnaires from previous studies^12^,^16^-^19^ were adapted for use in our study. A set of 30 questionnaire items was first generated from these studies. The items were constructed with dichotomous responses (yes/no) as well as a 5-point response scale (strongly agree, agree, neutral, disagree and strongly disagree). In order to assess content validity, the 30-item self-administered questionnaire was sent to three academics with skills in questionnaire design and psychometrics from the Clinical Pharmacy Department at the University of Uyo. These experts were requested to evaluate each of the items for clarity, relevance, and how well they reflected the study's objectives. Ultimately, nine items were dropped because they were either redundant or irrelevant, and two items which were considered ambiguous were modified. The resulting 21-item instrument was pretested with 10 students from the three programmes for readability, length, and comprehension.

Data were collected by both paper and electronic modes of self-administration. For the paper mode of administration, a trained research assistant in each of the participating universities was involved in data collection. The paper-based questionnaire was also administered to eligible students during a 2-day Nigerian Healthcare Students' Summit held on the 28th – 29th of November, 2019 at Bingham University, Abuja, Nigeria. Questionnaires were retrieved at the end of each day of the summit after ensuring that all fields were completed. Also, an online version of the questionnaire was created using Google form. The link to the questionnaire was shared via WhatsApp and email for students who did not attend the summit and hence, did not complete the paper-based questionnaire. A reminder was sent on a weekly basis via these platforms to increase the response rate. Data collection lasted for two months (i.e., November 28, 2019 to January 31, 2020).

### Statistical analysis

Data were analysed using the Statistical Product and Service Solutions (SPSS) IBM version 20.0. Frequencies and percentages were used to summarise participants' demographic data and responses. Answers to questions that used a 5- point Likert scale were merged into dichotomous categories (strongly agree/agree and strongly disagree/disagree/neutral). Pearson's Chi-square test (or Fisher's exact test where appropriate) was used to determine the association between categorical variables and compare the parameters as appropriate. All analyses were considered statistically significant at p < 0.05.

## Results

Three hundred and thirty-five students completed the questionnaire. A total of 167 students participated in the paper survey, while 168 students completed the electronic survey. However, four of the completed questionnaires (three from paper survey and one from online survey) were not included in the analysis due to errors and incompleteness. Three hundred and thirty-one completed questionnaires were used for final analysis.

Of the 331 students who participated in the survey, 180 (54.4%) were females. Mean age of respondents was 23±3 years; majority (208/331, 62.8%) were in the age range of 21-25 years. One hundred and fourteen (34.4%) were in their fifth year of study. Table 1 shows a summary of socio-demographic characteristics of respondents.

**Table 1:** Socio-Demographic Information of Respondents (N = 331)

Variable	Frequency	Percent
**Gender**		
Male	151	45.6
Female	180	54.4

**Age (years)**	22.6 ± 3.0*	
≤ 20	82	24.8
21-25	208	62.8
26-30	39	11.8
>30	2	0.6
**Geopolitical Zone of School**		
North Central	59	17.8
North West	58	17.5
North East	57	17.2
South South	57	17.2
South East	51	15.4
South West	49	14.8

**Course of Study**		
Medicine	110	33.2
Pharmacy	111	33.5
Nursing	110	33.2
**Year of Study**		
3rd Year	96	29.0
4th Year	96	29.0
5th Year	114	34.4
6th Year	25	7.6

* Mean ± standard deviation

### Knowledge and perceptions of antimicrobials and antimicrobial resistance

With regards to factors that promote AMR, majority (78.9%) of the students agreed that widespread use of antimicrobials promotes AMR. Less than half (21.1%) of the students were aware that poor hand hygiene promotes AMR. One hundred and fifty-two respondents (45.9%) agreed that AMR is promoted by substandard quality of antimicrobials; however, there was a significant difference between the three categories of healthcare students (42.7%, 37.3% and 57.7% for medicine, nursing and pharmacy, respectively, p = 0.007). Most (86.4%) of the respondents agreed that AMR leads to increased morbidity and health care costs. Majority (94.3%) perceived AMR as a worldwide problem; there was no significant difference in perception of prevalence of AMR between medicine, nursing and pharmacy students (p = 0.952). Among the study cohorts, only 11.8% did not perceive AMR as a significant problem in Nigeria with no significant difference between the three groups of students (p = 1.000).

Regarding factors that can combat AMR, 58.0% perceived the establishment of national AMR surveillance as one, with statistically significant difference between the groups (59.1%, 47.3% and 67.6% for medicine, nursing and pharmacy respectively, p = 0.009). Summary of Knowledge and perceptions of antimicrobials and AMR is provided in Table 2.

**Table 2:** Knowledge and perceptions of antimicrobials and antimicrobial resistance - percentage who agreed/strongly agreed to each statement

Item	All N =331	Medicine n = 110	Nursing n = 110	Pharmacy n = 111	*P*
Antibiotics refer to drugs that kill bacteria, whereas antimicrobials include drugs that kill viruses, fungi or bacteria	84.9%	85.5%	80.9%	88.3%	0.303
Antimicrobials kill both good and bad microbes	71.3%	76.4%	71.8%	65.8%	0.217
Antimicrobial resistance is a worldwide problem	94.3%	94.5%	94.5%	93.7%	0.952
Antimicrobial resistance leads to increased morbidity and health care costs.	86.4%	86.4%	85.5%	87.4%	0.916
Antimicrobial resistance is not a significant problem in Nigeria	11.8%	11.8%	11.8%	11.7%	1.000
Appropriate use of antimicrobials can cause antimicrobial resistance	16.0%	14.5%	18.2%	15.3%	0.740
Inappropriate use of antimicrobials can harm patients	89.4%	92.7%	89.1%	86.5%	0.317
Appropriate use of antimicrobials will reduce problems with antimicrobial resistant organisms	91.8%	90.9%	92.7%	91.9%	0.885
**Antimicrobial resistance is promoted by:**					
Widespread/overuse of antimicrobials	78.9%	82.7%	71.8%	82.0%	0.086
Prescribing broad spectrum antimicrobials when equally effective narrower spectrum antimicrobials are available.	61.9%	65.5%	53.6%	66.7%	0.089
Poor hand washing practice	21.1%	20.9%	15.5%	27.0%	0.108
Poor adherence to prescribed medication	71.0%	74.5%	63.6%	74.8%	0.114
Substandard quality of antimicrobials.	45.9%	42.7%	37.3%	57.7%	**0.007**
**Antimicrobial resistance can possibly be combated by:**	69.8%	70.9%	63.6%	74.8%	0.187
Establishment of antimicrobial usage policies	44.1%	45.5%	39.1%	47.7%	0.406
Reduction of antibiotic use.	58.0%	59.1%	47.3%	67.6%	**0.009**
Establishment of national antimicrobial resistance surveillance.	63.4%	68.2%	55.5%	66.7%	0.101
	78.2%	82.7%	72.7%	79.3%	0.189
Development of institutional guidelines for antimicrobial use					
Education on antimicrobial therapy					

Bold values are significant at *p* < 0.05.

### Knowledge of antimicrobial stewardship, goals and team composition

Among the study cohort, majority (60.8%) were not familiar with the term ‘antimicrobial stewardship’. However, majority of the students answered ‘yes’ to the key concepts of AMS which are appropriate selection (66.8%), appropriate dosing and route (66.8%) and appropriate duration (64.7%) of antimicrobial therapy. There was no statistically significant difference between medicine, nursing and pharmacy students (p > 0.05). Regarding knowledge of AMS goals, only 45 (13.6%) participants selected the correct options, with 10.9%, 14.5%, and 15.3% from medicine, nursing and pharmacy, respectively (p = 0.595). Similarly, less than half, 75 (22.7%) selected the correct options of AMS team composition (infectious disease physicians, infection control staff and clinical/hospital pharmacists), with no statistically significant difference between healthcare student groups (p = 0.488).

### Perception of education on antimicrobials and antimicrobial stewardship

Healthcare students' perception of education on antimicrobials and antimicrobial stewardship is summarised in Table 3. Although respondents in the different healthcare programmes indicated they have attended lectures on rational use of antibiotics (72.8%), when to start antibiotics (78.5%), how to select the correct dosing of antibiotics (66.5%) and how to select the right duration of treatment for specific infections (66.8%), over half (86.3%) had no formal training on AMS. All 331 (100%) respondents indicated that they need more training on AMS.

**Table 3:** Perception of education on antimicrobials and antimicrobial stewardship

Variable	All N=331	Medicine n = 110	Nursing n = 110	Pharmacy n =111	*P*
**Attended formal lectures that addressed the following topics:**					
Rational use of antibiotics in general	241 (72.8)	82 (74.5)	77 (70.0)	82 (73.9)	0.716
When to start antibiotics	260 (78.5)	85 (77.3)	85 (77.3)	90 (81.1)	0.728
How to select the correct dosing of antibiotics	220 (66.5)	78 (70.9)	71 (64.5)	71 (64.0)	0.480
How to select the right duration of treatment for specific infections.	221 (66.8)	75 (68.2)	74 (67.3)	72 (64.9)	0.864
**Rating of AMS knowledge**					
Poor	122 (36.9)	42 (38.2)	39 (35.5)	41 (36.9)	
Average	128 (38.7)	41 (37.3)	49 (44.5)	38 (34.2)	0.737
Good	70 (21.1)	23 (20.9)	19 (17.3)	28 (25.2)	
Very good	11 (3.3)	4 (3.6)	3 (2.7)	4 (3.6)	
**Had formal training on AMS**					
Yes	47 (13.7)	15 (13.6)	17 (15.5)	14 (12.6)	0.826
No	289 (86.3)	95 (86.4)	93 (84.5)	97 (87.4)	
**Would like additional**					
**training on AMS**					
Yes	331 (100.0)	110 (100.0)	110 (100.0)	111 (100.0)	–
No	0 (0.0)	0 (0.0)	0 (0.0)	0 (0.0)	

*AMS* = Antimicrobial stewardship; values are frequency (percent).

## Discussion

In Nigeria, newly inducted doctors, pharmacists and nurses are engaged in prescribing, dispensing antimicrobials, and caring for patients, respectively under the supervision of preceptors during their internship year, and during the mandatory one-year national service. The roles of doctors, pharmacists and nurses in antimicrobial stewardship and the impact of their intervention have been previously described.^8^, ^20^-^24^ Considering the importance of these professionals in the successful implementation of antimicrobial stewardship, the present study sought to evaluate healthcare students' knowledge and perceptions of antimicrobial resistance and stewardship in universities across the six geopolitical zones of Nigeria. Generally, students in this study had good knowledge of antimicrobials and factors which promote resistance, as well as programmes that can combat antimicrobial resistance. These findings are consistent with previous studies.^12^,^16^-^19^ However, majority of students were unaware of AMR burden in Nigeria. This finding highlights the need for national AMR surveillance system and educational campaign to create awareness on the problem of AMR among healthcare students and members of the public. Although majority of students in the different courses indicated they have attended lectures on appropriate use of antimicrobials, few were aware of the term ‘antimicrobial stewardship’, and even fewer had attended formal lecture on AMS. Furthermore, only a minority of participants correctly identified the goals of AMS and team composition (14% and 23%, respectively). These findings indicate a gap in the education and training of future healthcare professionals, and thus call for incorporation of AMS principles in medicine, pharmacy and nursing programmes in the country.

The World Health Organisation (WHO) identifies education and training of healthcare workers and students as an integral part of all AM containment activities,10 and in collaboration with Public Health England recently developed curricula guide to ensure adequate education and training.^25^ The goal of this guide is to ensure healthcare workers become good stewards of antimicrobials in whatever roles they perform by handling antimicrobials as a scarce and limited resource, following and adhering to evidence-based clinical guidelines when prescribing/dispensing antimicrobials and regular practice of infection prevention and control measures to prevent the spread of germs. To ensure Nigeria healthcare students are exposed to AMS principles as their counterparts in other parts of the world,^26^-^28^ healthcare faculties in Nigerian universities and health education regulatory bodies, including the Nigeria Medical Council, Pharmacists Council of Nigeria and Nursing & Midwifery Council of Nigeria should therefore review their curricula in line with the WHO guide. In addition, healthcare facilities in Nigeria should consider implementing AMS programmes to provide hands-on experience for newly inducted doctors, pharmacists and nurses during their internship year. Although a number of barriers prevent effective implementation of AMS programmes in resource-limited settings, including Africa,^29^ experience from other parts of the continent show that AMS can be implemented.^30^

### Strengths and limitations of study

The strength of this study is that it simultaneously evaluates knowledge and perception of healthcare students who would be directly involved in antimicrobial prescribing, dispensing and administration following completion of their studies. While there was no significant difference in knowledge and perception in majority of the items among the healthcare students surveyed, the study identified a gap in knowledge of antimicrobial stewardship principles that requires attention.

The limitations of this study include the fact that it relied on respondents' report and therefore subject to bias associated with self-completion questionnaire method. Additionally, the use of both paper and electronic questionnaires to survey students in this study could have influenced participants' response patterns. Nevertheless, only little differences in response patterns between self-administered paper questionnaires and self-administered electronic/on-line questionnaires have been reported.^31^

The convenience sample employed in selecting the respondents is another limitation. As a result, respondents might not be representative of the student population offering medicine, nursing and pharmacy in Nigeria. Nevertheless, this effect was minimized by the relatively large number and geographical spread of institutions from which students were surveyed. The study also focused on medical, pharmacy and nursing undergraduate students; thus, findings may not be generalised to other categories of healthcare students. In line with one-health approach of the WHO, a comparative study involving dentistry and veterinary students is planned.

## Conclusions

Students who participated in the study had good knowledge of antimicrobials, factors which promote resistance and programmes that can combat antimicrobial resistance. However, majority were neither aware of the term ‘antimicrobial stewardship’ nor had attended formal lecture on antimicrobial stewardship. Current undergraduate healthcare curriculum should be reviewed to incorporate antimicrobial stewardship principles. There should also be public awareness campaigns on antimicrobial resistance to create awareness among healthcare students.
